# Optimization Design of Haptic Units for Perception Feedback Interfaces Based on Vibrotactile Amplitude Modulation

**DOI:** 10.3390/biomimetics10090597

**Published:** 2025-09-07

**Authors:** Weichao Guo, Jingchen Huang, Lechuan Zhou, Yun Fang, Li Jiang, Xinjun Sheng

**Affiliations:** 1 Shanghai Key Laboratory of Intelligent Robotics, Meta Robotics Institute, Shanghai Jiao Tong University, Shanghai 200240, China; guoweichao90@gmail.com (W.G.); huang_jc@sjtu.edu.cn (J.H.); frivolity@sjtu.edu.cn (L.Z.); fangyun@sjtu.edu.cn (Y.F.); 2 State Key Laboratory of Mechanical System and Vibration, Shanghai Jiao Tong University, Shanghai 200240, China; 3 State Key Laboratory of Robotics and System, Harbin Institute of Technology, Harbin 150080, China

**Keywords:** tactile sensation, vibration units, mechanical crosstalk, joint optimization design, packaging materials, finite element analysis

## Abstract

Tactile sensation is a crucial sensory pathway for humans to acquire information from the environment, and vibration feedback is one form of tactile feedback, offering advantages such as low cost, ease of integration, and high comfort. Avoiding mechanical crosstalk without changing the spacing between vibration units is a significant challenge in the design of haptic interfaces. This work focuses on the joint optimization design of vibration source characteristics and packaging materials of vibration units. From a theoretical modeling perspective, we explore the correlation between material properties and the amplitude of vibrations generated on the skin surface. A three-layer vibration unit optimization design scheme using a pogo pin structure is thus proposed. Parameters are optimized through finite element analysis, and experimental results prove that the three-layer vibration unit with pogo pins has amplitude modulation capabilities, laying the foundation for the design of array-based vibration tactile feedback interfaces and human-inspired grasp control.

## 1. Introduction

Tactile sensation is a crucial sensory pathway for humans to acquire information from the environment, playing a central role in perception and feedback [1]. Mechanoreceptors in the skin play a central role in tactile signal transduction [2]. These specialized nerve endings convert mechanical stimuli—such as pressure, vibration, and deformation—into electrical signals that are conveyed through sensory pathways to the central nervous system. Owing to their dense and widespread distribution across the skin, the body’s largest organ, tactile receptors provide a rich channel of information [3]. This makes tactile sensation a promising modality for sensory substitution, particularly in cases where auditory or visual functions are impaired [4,5]. For instance, visually impaired individuals rely on fingertip contact with Braille to recognize letters, words, and sentences, thereby facilitating reading and learning. Likewise, hearing-impaired individuals employ vibrating alarms to receive tactile feedback in place of auditory cues, enabling activities such as waking or alert detection [6].

In the field of human–computer interaction, haptic feedback has emerged as a crucial mode of information transmission. Its applications span prosthetic limb control [7], remote social interaction [1], classroom teaching [8], surgical procedures, and virtual reality [9], all of which demonstrate its effectiveness in enhancing user experience. Compared with traditional single-modal control, human–machine interfaces that integrate haptic feedback allow users to execute more intricate and complex tasks with greater efficiency and precision, such as surgical manipulation [10] and grasp control [11,12].

Vibration feedback is one form of tactile feedback, offering advantages such as low cost, real-time response, ease of integration, and high comfort [13]. In practical applications, vibration interface devices encode information into vibrations with different timing, frequency, intensity, and position. The design of these interface devices directly affects the efficiency of information transmission and the user experience.

To improve the wearability of haptic interfaces, compact dimensions, lightweight construction, and low power consumption are essential design objectives for array-type vibration devices. In practice, these interfaces are commonly affixed to the skin using adhesive bonding or strapping. However, due to the viscoelastic nature of skin, vibrations generated by a single unit inevitably propagate to the surrounding area, stimulating tactile receptors beyond the intended site. This unintended spread is prone to mechanical crosstalk [14]. Avoiding mechanical crosstalk without changing the spacing between vibration units is a significant challenge in the design of haptic interfaces. Existing array-based vibration interface designs have attempted to address this issue by increasing the spacing between vibration units [15], adding feedback modules [9], or innovatively designing motors [16]. However, these methods are relatively complex and lead to larger interface sizes.

This work focuses on the joint optimization design of vibration source and encapsulation materials of vibration units. From a theoretical modeling perspective, we explore the correlation between material properties and the amplitude of vibrations at the edge of vibration unit generated on the skin surface. A three-layer vibration unit optimization design scheme using a pogo pin structure is proposed.

## 2. Materials and Methods

**Figure 1:**
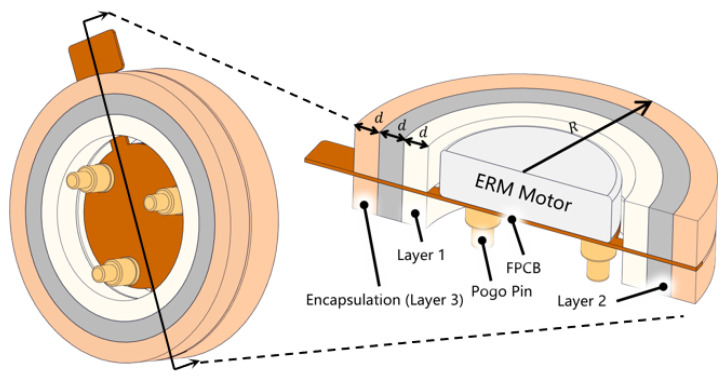
Schematic diagram of an eccentric rotating mass motor (ERM)-based vibration unit featuring three layers of encapsulation material and three evenly distributed pogo pins.

An eccentric rotating mass motor (ERM) installed in reverse configuration, with the motor soldered on a flexible printed circuit board (FPCB), is shown in Figure 1. Three pogo pins (PZ8116, Haitai Hongqiang, China) are soldered beneath the FPCB. The distance between the center of the pogo pin and the motor is 2.25 mm, the total length of the pogo pins is 2.4 mm, the travel of the pogo pin is 0.3 mm, and the spring constant of the pogo pin is 2942 N/m. This chapter elaborates the selection of parameters to be optimized and the design of simulation and experiments.

### 2.1. Structure of Vibration Unit

In existing array-based vibration interfaces, linear resonant actuators (LRAs), voice coil motors (VCMs), and ERMs are three commonly used vibration motors [17]. Research findings indicate that ERMs can efficiently induce stronger vibrations and are compact and easy to attach, making them an ideal choice for vibration sources in array-based haptic feedback interfaces [18]. Considering the miniaturization requirements of haptic units, this work selects an ERM motor with a height of 2 mm and a diameter of 7 mm (LCM0720A3463F, Leader, China) as the vibration source.

**Figure 2:**
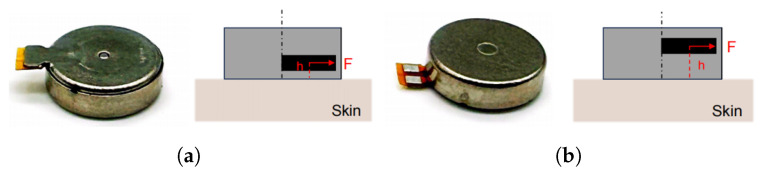
Photographs and schematic diagrams of the ERM in forward and reverse orientations. The yellow edges indicate the connections to the circuit board, the black blocks represent the eccentric rotor, h denotes the distance from the skin to the rotor center, and F indicates the direction of the centrifugal force. (**a**) ERM in forward orientation; (**b**) ERM in reverse orientation.

To address crosstalk between vibration units, we aim to optimize the vibration structure to minimize the amplitude at the edges and maximize the amplitude at the center. To reduce the amplitude at the edges, the adoption of a pogo pin structure is proposed as a means of altering the distribution of vibrations induced by the motor on the skin. To increase the amplitude in the center, two possible orientations are proposed, forward and reverse as shown in Figure 2, for simulation and testing.

### 2.2. Encapsulation Materials of Vibration Unit

Since the encapsulation material is in direct contact with the skin, its physical properties significantly influence the efficiency and attenuation patterns of vibration propagation. The goal of optimizing the encapsulation material design is to enable the vibration unit to modulate its amplitude, ensuring that the vibration waves generated by the motor rapidly attenuate once they reach the external boundary of the vibration unit. Four stretchable silicone materials including Ecoflex 00-10, Ecoflex 00-50, Dragon skin 10 Medium, and Dragon skin 30 (Smooth-On, Inc., Macungie, PA, USA) are selected to test the influence of encapsulation material.

**Figure 3:**
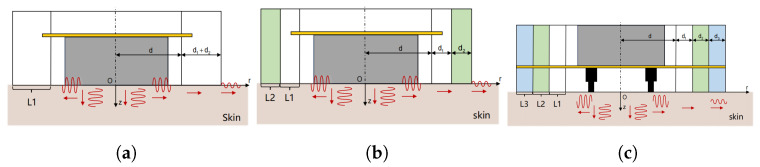
Three vibration units with different structural configurations, where L1, L2, and L3 denote the first, second, and third encapsulation layers, respectively. The yellow horizontal layers represent the flexible printed circuit board (FPCB). (**a**) forward-oriented ERM with one layer of encapsulation; (**b**) forward-oriented ERM with two layer of encapsulation; (**c**) reverse-oriented ERM with three layers of encapsulation and pogo pins.

The number of encapsulation layers, along with possible combinations and stacking order, also modulates the amplitude at the edge of the vibration unit. Three configurations of vibration unit are shown in Figure 3: single-layer, double-layer, and 3-layer unit with pogo pin.

### 2.3. Design of Simulation

**Figure 4:**
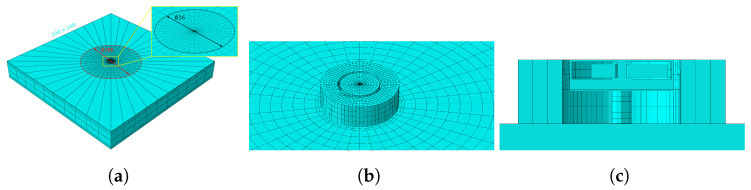
Finite element model in ABAQUS. (**a**) mesh generation for the forearm with a four-layer structure, where the zoomed area represents the “near-field” with a mesh spacing of 0.5 mm, the red circle represents the field with a mesh spacing of 2mm; (**b**) modeling of a three-layer vibration unit with reversed ERM and pogo pins attached to the simulated forearm; (**c**) cross-sections of the vibration unit.

To simulate the interaction between vibration unite and human skin, a four-layer forearm model was created in ABAQUS, as shown in Figure 4a. The model parameters are listed in Table 1. The forearm was set as a rectangular parallel-piped model with dimensions of 240 mm × 240 mm × 31.9 mm. To simulate the continuity of human skin and tissue and avoid vibration echoes caused by the boundaries of the finite element model, which could interfere with the calculation results, the model consists of two parts: a finite element mesh and an infinite element mesh. The mesh inside the 100 mm diameter red circle shown in Figure 4a is the finite element mesh, and that outside is the infinite element mesh. The finite element mesh uses eight-node three-dimensional solid elements (C3D8 elements), with each node having three degrees of freedom. The infinite element mesh uses eight-node three-dimensional infinite elements (CIN3D8 elements).

During the simulation, the vibration source was placed at the center of the model shown in Figure 4b. The mesh shape was set to radial from the center to the periphery given that vibration propagates radially outward and was symmetrical along the circumference. Taking into account both model accuracy and computational efficiency, the area within 8 mm of the model center was considered near-field, with a smaller mesh spacing of 0.5 mm. Within the range of 8 mm to 50 mm from the model center, the mesh spacing was increased to 2 mm. When setting the mesh, the element shape should be as regular as possible to avoid element distortion or spikes. Tie constraints are used between layers of the four-layer forearm model to bind adjacent surfaces together, ensuring that the nodes on these surfaces maintain a relatively fixed position. Boundary conditions are set on the lower surface of the skeleton layer to fix its displacement in the x, y, and z directions, simulating a stationary arm. The parameter settings for each layer of simulated forearm and their respective contact constraint are shown in Table 1.

To model the behavior of pogo pins within vibration units, the pogo pins were defined as the vibration source. The eccentric rotating mass (ERM) motor was inverted and affixed to the flexible circuit board using tie constraints, thereby simulating the mechanical interaction between the motor and the circuit board. Additionally, tie constraints were applied to secure three pogo pins beneath the flexible circuit board, representing the soldered connections between the pins and the board. The interaction between the pogo pins and the skin was modeled as frictionless hard contact.

Layers of encapsulation materials are simulated as hollow cylinders with different modulus of elasticity, density and Poisson’s ratio. For example, to simulate a vibration unit with three layers, three 1mm thick hollow cylinders are placed outside the vibration source, with tie constraints set between the layers. Tie constraints are also added between the bottom surfaces of the three hollow cylinders and the skin surface. The solver was set to Dynamic Implicit, and the solver time step is set to 0.05 ms. An angular displacement of 120 revolutions per second was applied to the eccentric mass of the vibration motor. The vibration amplitude of the skin surface outside the vibration unit was calculated to examine the effects of different design parameters on the amplitude modulation capability of the vibration unit.

### 2.4. Design of Experiment

**Figure 5:**
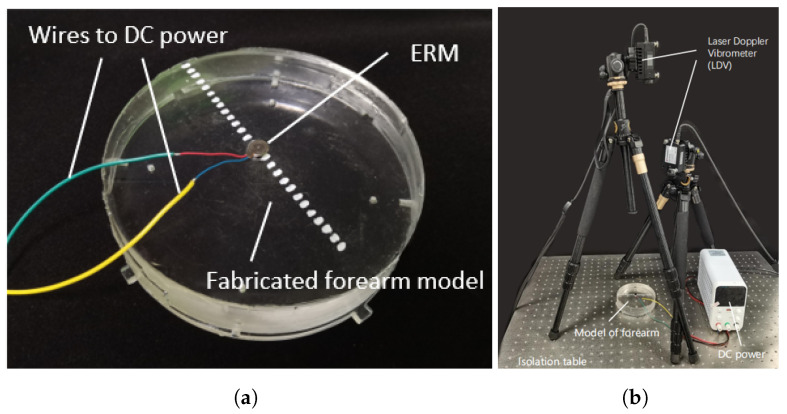
Experimental setup for validating simulation accuracy. (**a**) Photograph of the transparent forearm model with an ERM affixed at the center where the white markers are used to indicate vibration on the skin surface; (**b**) photograph of the experimental platform, where the ERM is driven by a DC generator and two Doppler laser vibrometers (DLVs) oriented toward the forearm model.

To validate the accuracy of the finite element model, simulations were performed in ABAQUS to analyze vibrations induced by a motor affixed to the skin surface, complemented by experiment with same configuration on a vibration measurement platform with two Doppler laser vibrometers as is shown in Figure 5. Based on the parameters listed in Table 1, a forearm model was fabricated by adjusting the ratio of transparent silicone (Model: 9300/9310/9400, Manufacturer: Hongye, Shenzhen, China). The epidermis, dermis, and muscle layers were cast using a mixture of silicones 9310 and 9300 at a ratio of 1:1.05, while the subcutaneous layer was formed from 9400 silicone and the skeletal layer from acrylic. An eccentric rotor vibration motor was mounted upright at the center of the silicone model using double-sided adhesive tape (Model: DSP9889, Manufacturer: Dongxin, Hangzhou, China) and driven by a 3.3 V DC voltage source. Vibration responses at different surface locations were then recorded using two Doppler laser vibrometers (Model: KVD-4525L, Manufacturer: KathMatic, Nanjing, China).

**Figure 6:**
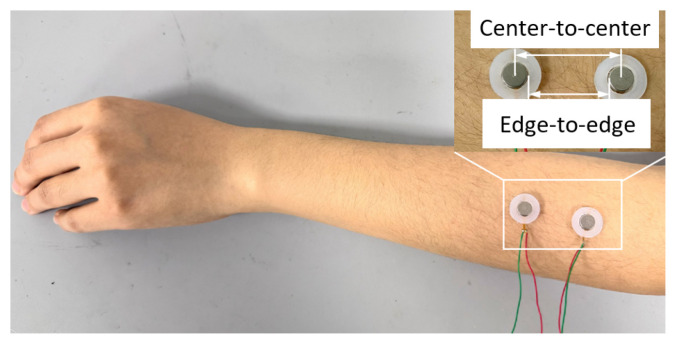
Experimental setup for two-point discrimination threshold measurement.

To validate the effectiveness of the optimized design of the vibration unit, a two-point discrimination threshold [19] experiment was performed. The two-point discrimination thresholds were measured for the single vibration motor, the double-layer wrapped vibration unit, and the three-layer vibration unit with pogo pins, under both simultaneous and successive stimuli modes [20] on the forearm. A total of 10 healthy volunteers, including 6 males and 4 females with an average age of 24.8 ± 2.70 years, participated in the experiment. Two identical vibration units were fixed at the one-third position of the back of the forearm near the elbow joint, as shown in Figure 6. The experimental design and execution strictly adhered to the standards set by the Declaration of Helsinki and were approved by the ethics committee of Shanghai Jiao Tong University (approval number: E20230257I).

## 3. Results and Discussion

### 3.1. Validation of the Forearm Finite Element Model

**Figure 7:**
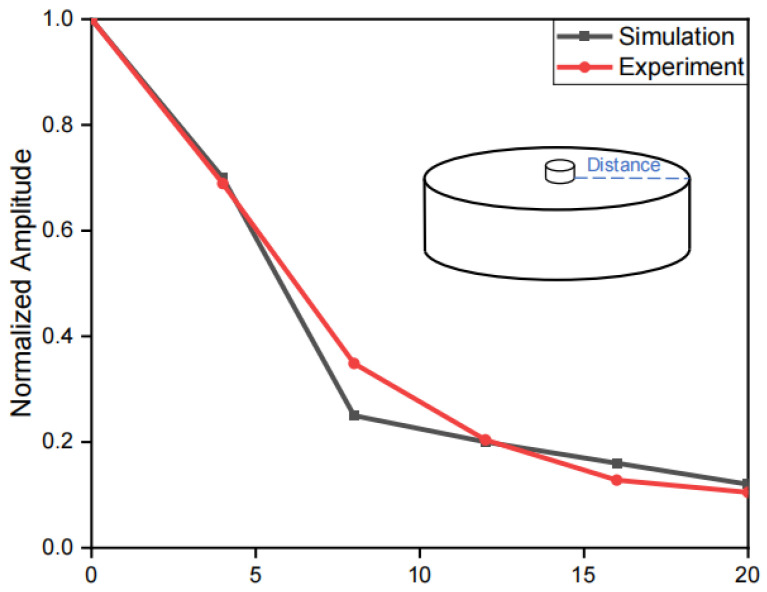
Comparison between simulation and experimental results of an ERM motor affixed on the forearm.

The comparison of simulation and experimental results of the vibration amplitude stimulated on the forearm is presented in Figure 7. During the experiment, the vibration motor oscillated at a frequency of 100–150 Hz. The sampling frequency of the laser profilometer was set to 2 kHz. The acquired data underwent filtering through a Butterworth filter with a frequency range of 40 Hz to 200 Hz, coupled with a 50 Hz comb filter, to eliminate interference from low-frequency noise and mains power frequency noise.

The 0 mm position denotes the motor edge. Along the forearm model, results from ABAQUS simulations and experimental measurements were examined every 4 mm from 0 mm to 20 mm radially from the motor edge. The curve’s fitting quality was assessed by calculating the coefficient of determination $R^{2}=0.9831$, which suggests a high fitting quality who supports further optimization of structure and material parameters via ABAQUS.

### 3.2. Finite Element Simulation on Structure of Vibration Unit

**Figure 8:**
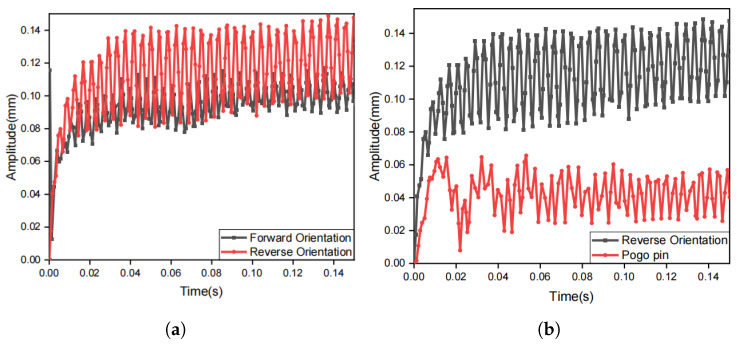
Simulation result on different structures of vibration unit. (**a**) amplitude-time plot at the center of vibration unit for forward and reverse orientations; (**b**) amplitude-time plot at the edge of vibration unit for reverse orientation and reverse orientation with pogo pins.

Finite element analysis and simulation experiments confirm that the reverse orientation of the motor produces a greater vibration amplitude in the center of the vibration unit compared to the forward orientation as shown in Figure 8a. Simulations also confirm that the pogo pin structure can effectively reduce the vibration amplitude at the edge of the vibration unit to half of its original value, as shown in Figure 8b.

### 3.3. Finite Element Simulation on Encapsulation Materials

**Figure 9:**
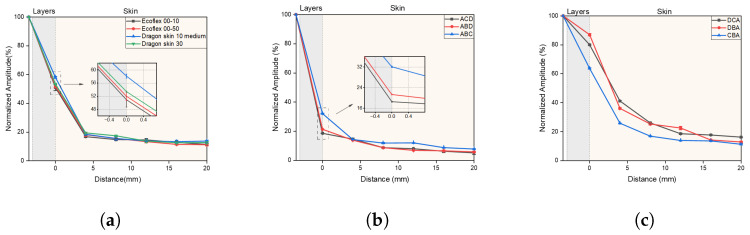
Normalized amplitude-distance plot for different encapsulation materials. The 0 mm position denotes the vibration unit edge. “A” stands for Ecoflex 00-10, “B” for Ecoflex 00-50, “C” for Dragon skin 10 medium and “D” for Dragon skin 30. (**a**) single-layer vibration units; (**b**) triple-layer encapsulated vibration units that restrain vibration; (**c**) triple-layer vibration units that enhance vibration.

Figure 9 shows the amplitude generated by different vibration units on the skin as a function of position. Compared to a single-layer encapsulation configuration, the simulation results demonstrate that the three-layer vibration unit has a high potential in amplitude modulation, both in restraining vibration and in enhancing vibration. In the case of the single-layer vibration unit, Figure 9a, the normalized amplitude at the edge ranged between 50% and 58%, serving as a reference for the modulation effect. The use of triple-layer encapsulations can significantly restrict the normalized amplitude, reducing it to a minimum of 19% at the edge, as is shown in Figure 9b. This improvement was achieved by employing Ecoflex 00-10, Dragon Skin 10 Medium, and Dragon Skin 30 as the first, second, and third layers, respectively. In contrast, the selection of layers presented in Figure 9c can increase the normalized amplitude at the edge to a maximum of 90%.

**Figure 10:**
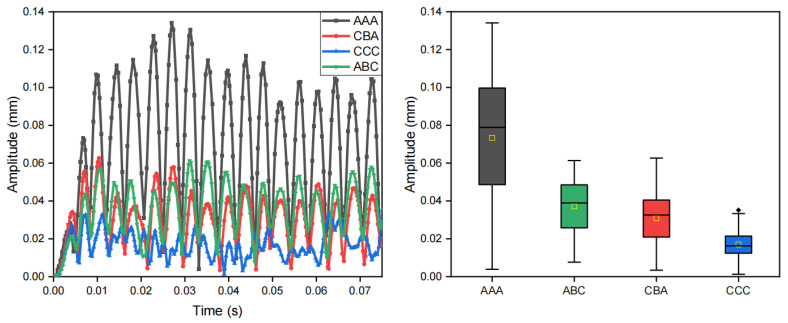
Effect of encapsulation material density on vibration modulation performance. The plot on the left is the displacement–time plot at the edge of the vibration unit where “A”, “B”, “C” correspond to the parameter configurations in Table 2. The plot on the right is the corresponding box plot.

**Figure 11:**
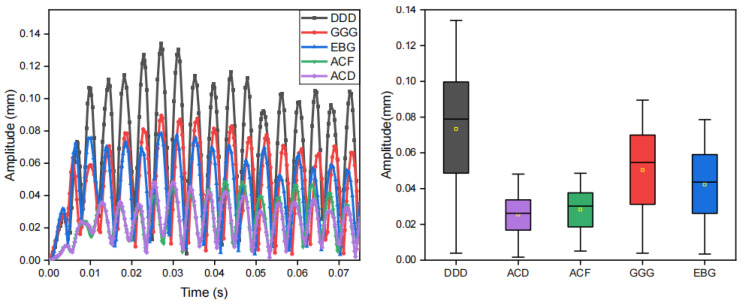
Effect of encapsulation material elasticity on vibration modulation performance. The plot on the left is the displacement–time plot at the edge of the vibration unit where “A”, “B”, “C”, “D”, “E”, “F”, “G” correspond to the parameter configurations in Table 3.

**Figure 12:**
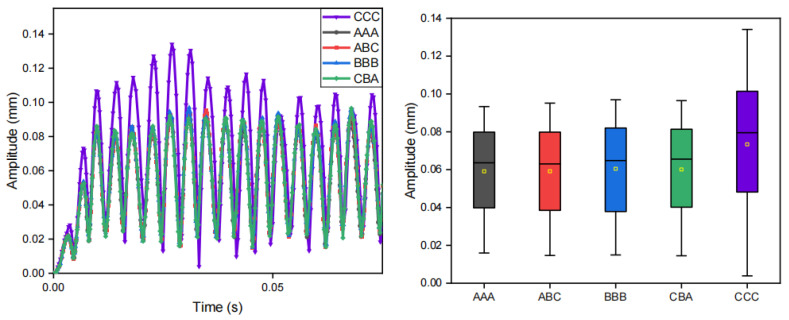
Effect of encapsulation material Poisson’s ratio on vibration modulation performance. The plot on the left is the displacement–time plot at the edge of the vibration unit where “A”, “B”, “C” correspond to the parameter configurations in Table 4.

Using the finite element method, we quantitatively evaluated the specific impacts of three key parameters of the encapsulation material on the amplitude modulation capability: density, modulus of elasticity, and Poisson’s ratio. The influence on vibration modulation of each parameter and their respective material configuration are presented in Figure 10, Figure 11 and Figure 12, Table 2, Table 3 and Table 4, respectively.

These simulations suggest that an optimization of encapsulation materials parameter can improve the modulation of vibration. Among the parameters, the density of encapsulation material has a more significant influence.

### 3.4. Two-Point Discrimination Threshold Experiment

Based on the optimal vibration unit structure and encapsulation materials identified through simulation, prototype units were fabricated incorporating the most effective modulation configurations. Three designs were selected for evaluation in the two-point discrimination threshold experiment: (1) an ERM in forward orientation without encapsulation, serving as the reference; (2) a double-layer vibration unit in forward orientation, with Ecoflex 00-10 as layer 1 and Dragon Skin 30 as layer 2 and (3) a triple-layer vibration unit in reverse orientation with pogo pin connection, employing Ecoflex 00-10, Dragon Skin 10 Medium, and Dragon Skin 30 as layers 1, 2, and 3, respectively.

**Figure 13:**
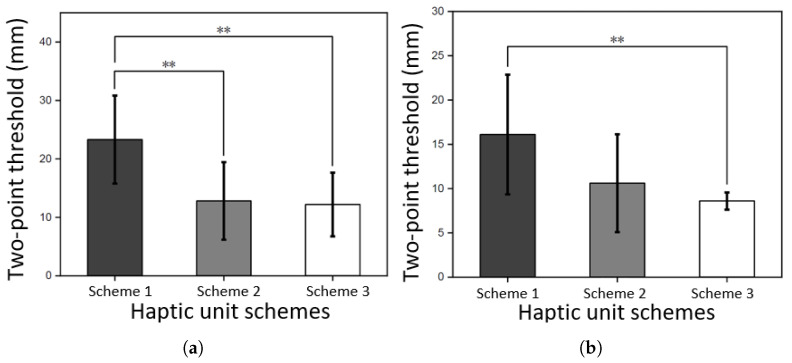
Edge-to-edge two-point threshold measurement results. Schemes 1, 2, and 3 are the results of an ERM vibration unit with no encapsulation in forward orientation, a double-layer encapsulation vibration unit in forward orientation, and a triple-layer vibration unit in reverse orientation with pogo pins, respectively. ** denotes *p* < 0.01. (**a**) results with simultaneous stimuli modes; (**b**) results with successive stimuli modes.

The results of two-point discrimination threshold experiment showed that the optimized design of the vibration unit significantly reduced the two-point discrimination threshold (*p* < 0.01), as shown in Figure 13. Under successive stimuli, the two-point discrimination threshold for the optimized vibration unit design is 8.6 mm. Compared to the state-of-the-art report [15], this design reduced the spacing between vibration units by 34% (from 13 mm to 8.6 mm). This result confirms the effectiveness of the optimized design in reducing mechanical crosstalk from the perspective of user’s tactile perception.

## 4. Conclusions and Future Work

### 4.1. Conclusions

This paper presents an optimization design of vibration units from two perspectives, namely, the structure of the vibration unit and the properties of its encapsulation materials. To ensure the reliability of the simulation settings, a laser Doppler vibrometer (LDV) experiment was conducted, which verified the accuracy of the ABAQUS modeling parameters. On this foundation, two sets of finite element analyses were carried out: one focusing on optimizing the structural configuration of the vibration unit, and the other on determining suitable material parameters for the encapsulation layers. The optimized design was further validated through threshold measurement experiments, which confirmed the predicted modulation performance. The results demonstrate that the three-layer vibration unit in reverse orientation with pogo pins achieves effective amplitude modulation, thereby laying a solid foundation for the development of array-based vibration tactile feedback interfaces and contributing to human-inspired grasp control strategies.

### 4.2. Future Directions

Building upon the current vibration unit design, future research may focus on several directions to further enhance the distinguishability of tactile feedback and thereby improve transmission efficiency and precision in complex tasks such as surgical manipulation and human-inspired grasp control: (1) development of array-based vibration interfaces; (2) encoding strategies for information transmission; (3) modulation of acceleration and spectral optimization.

## Figures and Tables

**Table 1:** Parameter settings of the forearm model in ABAQUS.

Layer	Thickness (mm)	Modulus of Elasticity (kPa)	Density (kg/m^3^)	Poisson’s Ratio
Epidermis and dermis	1.9	49	1116	0.47
Subcutaneous tissue	14	10	1116	0.47
Muscle	21	53	1116	0.47
skeleton	5	2,940,000	2000	0.32

**Table 2:** Density-dependent material parameter configuration in ABAQUS for triple-layer haptic units.

Layer	Density (kg/m^3^)	Modulus of Elasticity (kPa)	Poisson’s Ratio
A	1000	355	0.47
B	2000	355	0.47
C	4000	355	0.47

**Table 3:** Modulus of elasticity-dependent material parameter configuration in ABAQUS for triple-layer haptic units.

Layer	Density (kg/m^3^)	Modulus of Elasticity (kPa)	Poisson’s Ratio
A	1000	15	0.47
B	1000	100	0.47
C	1000	268	0.47
D	1000	355	0.47
E	1000	900	0.47
F	1000	1200	0.47
G	1000	3600	0.47

**Table 4:** Poisson’s ratio-dependent material parameter configuration in ABAQUS for triple-layer haptic units.

Layer	Density (kg/m^3^)	Modulus of Elasticity (kPa)	Poisson’s Ratio
A	1000	355	0.2
B	1000	355	0.33
C	1000	355	0.47

## Data Availability

The data and code of the current study can be obtained from the author upon reasonable request.
